# Spatial Distribution of Excitatory Synapses on the Dendrites of Ganglion Cells in the Mouse Retina

**DOI:** 10.1371/journal.pone.0086159

**Published:** 2014-01-17

**Authors:** Yin-Peng Chen, Chuan-Chin Chiao

**Affiliations:** 1 Institute of Systems Neuroscience, National Tsing Hua University, Hsinchu, Taiwan; 2 Department of Life Science, National Tsing Hua University, Hsinchu, Taiwan; Virginia Tech Carilion Research Institute, United States of America

## Abstract

Excitatory glutamatergic inputs from bipolar cells affect the physiological properties of ganglion cells in the mammalian retina. The spatial distribution of these excitatory synapses on the dendrites of retinal ganglion cells thus may shape their distinct functions. To visualize the spatial pattern of excitatory glutamatergic input into the ganglion cells in the mouse retina, particle-mediated gene transfer of plasmids expressing postsynaptic density 95-green fluorescent fusion protein (PSD95-GFP) was used to label the excitatory synapses. Despite wide variation in the size and morphology of the retinal ganglion cells, the expression of PSD95 puncta was found to follow two general rules. Firstly, the PSD95 puncta are regularly spaced, at 1–2 µm intervals, along the dendrites, whereby the presence of an excitatory synapse creates an exclusion zone that rules out the presence of other glutamatergic synaptic inputs. Secondly, the spatial distribution of PSD95 puncta on the dendrites of diverse retinal ganglion cells are similar in that the number of excitatory synapses appears to be less on primary dendrites and to increase to a plateau on higher branch order dendrites. These observations suggest that synaptogenesis is spatially regulated along the dendritic segments and that the number of synaptic contacts is relatively constant beyond the primary dendrites. Interestingly, we also found that the linear puncta density is slightly higher in large cells than in small cells. This may suggest that retinal ganglion cells with a large dendritic field tend to show an increased connectivity of excitatory synapses that makes up for their reduced dendrite density. Mapping the spatial distribution pattern of the excitatory synapses on retinal ganglion cells thus provides explicit structural information that is essential for our understanding of how excitatory glutamatergic inputs shape neuronal responses.

## Introduction

Retinal ganglion cells (RGCs) have diverse dendritic morphologies and distinct receptive field properties. They receive excitatory inputs from bipolar cells via glutamatergic synapses, while also receiving inhibitory inputs from amacrine cells via GABAergic and glycinergic synapses. Together these excitatory and inhibitory synapses determine the firing patterns of RGCs and how information is transferred from the eye to the brain [1]. Aside from the contribution of inhibitory synapses, it has been suggested that the distinct spatial patterns of excitatory synaptic input to dendrites of RGCs may characterize their functionally type. Following the lead of Jakobs et al. [2] in studying the rabbit retina, the present study examines the spatial distribution of glutamatergic synapses on the dendrites of a subset of RGCs in the mouse retina in order to reveal the correlation between synaptic spacing/distribution and their distinct dendritic morphologies.

To characterize the spatial pattern of synaptic inputs in RGCs, several techniques have been used previously to address this issue. For example, serial section electron microscopy has been used to study the synaptic connections of alpha cells and beta cells in cats [3]–[6], and the midget cells, parasol cells, and blue-ON cells in macaque monkeys [7]–[11]. In addition, light microscopy, in conjunction with immunocytochemistry of synaptic markers and microinjection of fluorescence dyes, has been used to examine the synaptic contacts on dendrites of various RGCs in marmoset monkeys [12]–[15], rabbits [16], [17], and guinea pigs [18]. However, these early attempts suffer from a number of shortcomings. For example, the electron microscopy studies are all based on incomplete reconstructions of a small number of RGCs, and the light microscopy studies lack the spatial resolution to ascertain the location of synaptic markers on the dendritic segments of RGCs, thus it was difficult to know if a punctum, representing a presynaptic protein, was on the ganglion cell membrane, or just somewhere in the vicinity of the ganglion cell.

One exception to the above approaches is a recent study of synaptic connections between starburst amacrine cells and direction selective ganglion cells using serial block-face electron microscopy in the mouse retina, in which they unambiguously showed that a synaptic asymmetry contributes to the computation of direction selectivity [19].

Recent progress in short-term organotypic retina culture and particle-mediated gene transfer has allowed the correct introduction and expression of fluorescently labeled synaptic markers in targeted cells [20], such as the glutamatergic synapses on dendrites of individual RGCs; this approach can be used to unambiguously visualize and quantify these cells [2], [21]–[23]. This is achieved by expressing postsynaptic density 95 (PSD95), a protein that is associated with most types of glutamate receptors [24]–[27], on the dendrites of various RGCs where the glutamatergic inputs from bipolar cells are located. By examining the spatial pattern of PSD95-GFP puncta on different types of RGCs in the rabbit retina, it has been demonstrated that the linear density of the excitatory inputs of various RGC types shows little specialization and their excitatory synapses are apparently spaced regularly, at 2–3 µm intervals, along the dendrites [2], [23].

In comparison with RGCs in the rabbit retina, the number of RGC types and their dendritic morphologies are much complex in the mouse retina. It has been estimated that at least 17–22 distinct types of mouse RGCs that are able to be distinguished morphologically [28]–[32]. However, their corresponding physiological properties and receptive fields have not been examined systematically. To investigate if these functionally different types of RGCs are determined in part by their spatial patterns of excitatory synaptic inputs, the aim of the present study was to correlate the spatial distribution of glutamatergic synapses with their distinct dendritic morphologies. Despite the fact that RGCs show a wide variety of sizes, morphologies, and functional complexity, we derived a single set of rules to describe the distribution of excitatory synaptic inputs in various types of mouse RGCs. These rules are similar to those found in the rabbit [2], [23], which suggests that there is likely to be a generic spatial pattern of excitatory synapses in mammalian RGCs.

## Materials and Methods

### Ethics statement

All procedures were approved by the Institutional Animal Care and Use Committee of the National Tsing Hua University and were in accordance with the ARVO Statement for Use of Animals in Ophthalmic and Vision Research.

### Retina preparation

Total of 7 adult C57BL/6 mice of either sex (age of 2–6 months) were used in the present study. Animals were anesthetized with an intraperitoneal injection of ketamine (10 mg/kg) and xylazine (10 mg/kg), and the eyeballs were enucleated with surgical scissors under a room light. After hemisection along the ora serrata, the lenses and vitreous humors were immediately removed. The posterior eyecups were then immersed in the oxygenated (95% O_2_ and 5% CO_2_) Ames’s medium (Sigma-Aldrich, St. Louise, MS) containing 23 mM NaHCO_3_. The retina was gently detached from the retinal pigment epithelium. Four radial cuts were made to facilitate flattening of the isolated retina. The mouse retinas were placed ganglion cell side up on a 0.4-µm Millicell tissue culture insert (Millipore, Bedford, MA), and gentle suction was applied to the tissue to allow proper attachment to the membrane.

### Particle-mediated acute gene transfer of PSD95-GFP

The expression plasmid pPSD95-GFP was a kind gift of Dr. Amane Koizumi (National Institute for Physiological Sciences, Okazaki, Japan). It allows the expression of a fusion protein of full-length PSD95 and GFP with the C-terminus of PSD95 linked via three glycine residues (introduced in the cloning process) to the N-terminus of GFP (for details, see Arnold and Clapham, 1999) [33]. Retinal ganglion cells were transfected via particle-mediated gene transfer in vitro using the Helios Gene Gun System (BioRad, Hercules, CA). The ratio of DNA to the gold particle (mean diameter 1.6 µm) was 1.5 µg plasmid/1 mg gold. The gold bullets were propelled into the tissue using pressurized helium at 90–100 psi.

### Organotypic culture of retina

Adult mouse retinal culture was performed based on the protocol for interphase culture of mammalian retinas [20], [22], [34], [35] with slight modifications. In brief, after particle-mediated acute gene transfer of PSD95-GFP, the retina-attached tissue culture insert was placed in a deep cell culture dish (Thermo Scientific, Waltham, MA), containing 20 mL Ames’ medium with 1% N-2 100X supplement, 1% penicillin/streptomycin/L-glutamine 100X solution, and 1% horse serum (Invitrogen, Carlsbad, CA). The retina was in contact with the medium via the Millicell filter on the photoreceptor side, and exposed to the incubator atmosphere (5% CO_2_, 37°C, humidified) on the ganglion cell side. To allow full expression of PSD95-GFP, the retina was cultured for three days, and the culture medium was changed daily.

### Retina fixation

The transfected and cultured retina was fixed with 4% paraformaldehyde (Electron Microscope Sciences, Hatfield, PA) and 0.1% glutaraldehyde (Sigma-Aldrich) in 0.1 M PB for one hour. The tissue was then rinsed with 0.1 M PB three times, 10 min each time. Finally, the retina was mounted ganglion cell side up in mounting medium containing DAPI (Vector Laboratories, Burlingame, CA).

### Image acquisition

All images (spatial resolution of 1024×1024) were acquired using a laser-scanning confocal microscope (LSM510, Zeiss, Germany) with a 100X oil-immersion objective lens (Plan Apochromat, 1.4 NA, Zeiss). For each cell examined, several z-stack images (z depth resolution of 1 µm, overlap 0.3 µm) were taken from the focal plane of the RGC axon to the inner nuclear layer (INL) to reveal the detailed dendritic morphology and the stratification level in the inner plexiform layer (IPL).

### Classification of PSD95-GFP expressed RGCs

The dendritic morphology and location of PSD95 puncta of each ganglion cell were reconstructed and digitized by NeuroLucida (MicroBrightField, Williston, VT). The traced cells were further analyzed by NeuroExplorer (MicroBrightField). To classify these PSD95-GFP expressed RGCs, five critical morphometric parameters were used to identify the cell types, including: (1) the soma diameter, (2) the dendritic field diameter, (3) the stratification level in the IPL, (4) the dendritic arbor density, and (5) the asymmetry index of dendrites at the soma (*Asymmetry index  =  [dendritic length difference between 1st and 3rd quarters + dendritic length difference between of 2nd and 4th quarters]/total dendritic length*). All mouse RGCs were classified into previously identified cell types (Völgyi et al., 2009).

### Data analysis

To characterize the spatial distribution pattern of the PSD95 puncta on the dendrite of each RGC, four analyses modified from previous studies [2], [23] were applied. In brief, these are: (1) the interval between puncta, which was measured as the distance between any two puncta along the dendrites within a dendritic segment, (2) the nearest-neighbor distance, which was calculated as the distance between any punctum and its nearest neighbor punctum along the dendrite, (3) the density of puncta as a function of dendritic branch order, which was obtained by dividing the number of puncta by the corresponded dendritic segment length at each branch order, and (4) the density of puncta as a function of distance from the soma (or, the Sholl analysis), which was computed by dividing the total puncta number by the total dendritic segment length within each 5 µm concentric ring.

In addition, to compare the spatial distribution patterns of PSD95 puncta across the different types of RGCs, the linear and areal puncta densities, as well as the dendritic arbor density, of each RGC were plotted against its dendritic field diameter. Furthermore, to examine the distribution of excitatory inputs on the proximal and distal dendrites across the different types of RGCs, the PSD95 puncta numbers and their cumulative puncta numbers were plotted against the distance from the soma (similar to the Sholl analysis above).

## Results

### Identification of PSD95-GFP expressed ganglion cells and the locations of excitatory synapses

**Figure 1:**
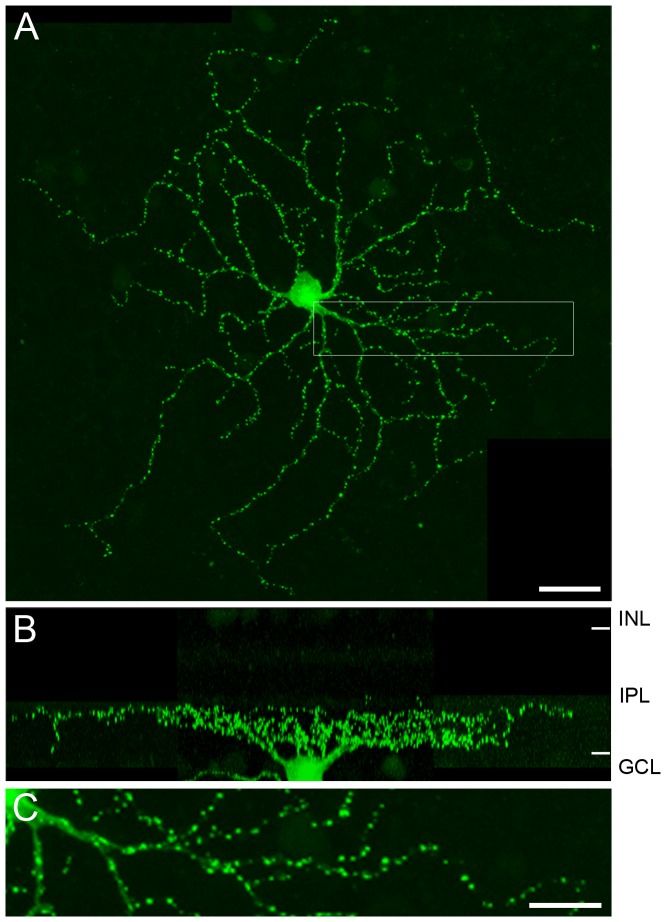
PSD95-GFP expression in the mouse RGC. (A) The PSD95-GFP puncta distributed along the dendrites of a G6 ganglion cell. This montaged confocal image was assembled from three high-resolution images taken by a 100X oil-immersion objective. (B) Vertical projection of the same cell showing the depth of dendritic stratification in the IPL. (C) Magnified view of the area boxed in (A). A part of soma is visible at the left, and the synaptic zones labeled by PSD95-GFP are shown as puncta of high pixel intensity on the dendrites. INL, the inner nuclear layer; IPL, the inner plexiform layer; GCL, the ganglion cell layer. Scale bar, 20 µm in (A) and 10 µm in (C).

Mouse RGCs were transfected with plasmids carrying PSD95-GFP using a gene gun. The resultant fluorescence images showed brightly labeled puncta distributed along the dendritic arbors (Fig. 1). The dendritic morphology was also revealed as a much fainter background labeling, which presumably arises from PSD95-GFP in transit to the puncta. This observation is in agreement with earlier studies involving the culturing of hippocampal neurons [25] and RGCs [2], [21], [22]. The pattern of labeling was consistent across all transfected cells. Previous studies have also confirmed that the PSD95-GFP puncta are in close apposition to known presynaptic (Kif3a or RIBEYE) and postsynaptic structures (AMPA glutamate receptors) in mammalian retinas [2], [21], [22], These findings support the idea that the PSD95-GFP puncta are a reliable means of identifying the sites of excitatory synapses on the RGCs.

**Figure 2:**
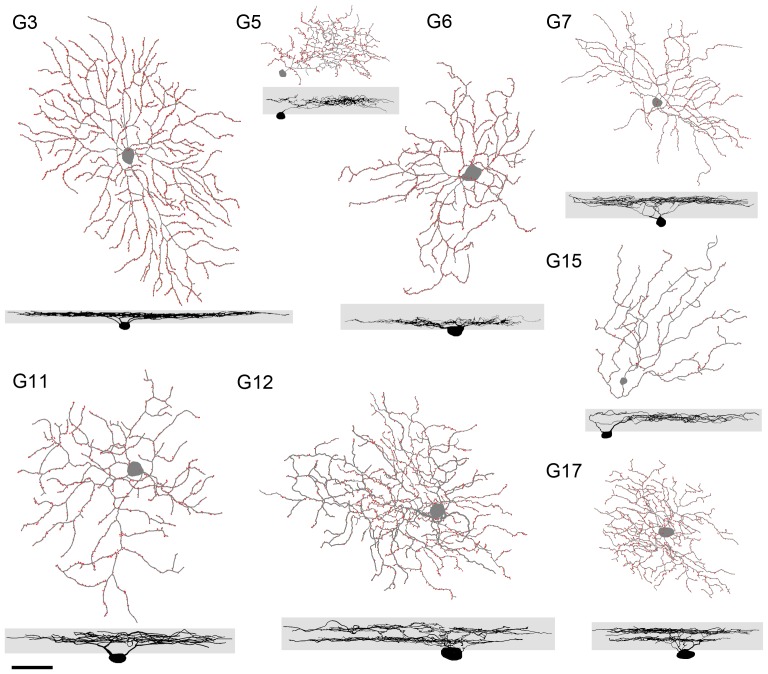
Synaptic input zones are uniformly distributed along the dendrites of most types of ganglion cells. The NeuroLucida drawings in the upper panel are the dendritic morphologies of the G3, G5, G6, G7, G11, G12, G15, and G17 types of mouse RGCs characterized in the present study. The excitatory glutamatergic inputs (PSD95-GFP puncta) are indicated by red dots. Vertical projection of the same cells in the lower panel shows the depth of dendritic stratification in the inner plexiform layer (shaded area). Scale bar, 20 µm.

We concentrated on 16 ganglion cells that had strong fluorescence signals and complete dendritic morphologies in order to carry out a quantitative analysis. These cells were digitized using the NeuroLucida software package to reveal their dendritic trees and PSD95 puncta (Fig. 2). Five morphometric parameters (see Methods for details) were used to classify these ganglion cells into eight previously identified cell types (Table 1). These were G3, G5, G6, G7, G11, G12, G15, and G17 ganglion cells [32], which represent a wide range of physiological and structural properties. For example, G3 cells are the OFF alpha ganglion cells [36]–[38], G12 cells are large bistratified ganglion cells [39], G15 cells are the OFF type of direction selective ganglion cell [40], [41], and G17 cells are the ON-OFF type of direction selective ganglion cell [39], [42]–[44].

**Table 1:** Morphological features of the PSD95-GFP expressed mouse ganglion cells chosen for analysis.

Cell type	#	Soma size (µm)	Dendritic field diameter (µm)	Stratification level in the IPL (%)	Dendritic arbor density (µm/µm^2^)	Asymmetry index	Number of PSD95 puncta	Linear puncta density (#/µm)	Equivalent ganglion cell classification			
									I	II	III	IV
G3	1	19.5	328.4	25±6	0.08	0.05	2835	0.41	A2_o_	Mon.cluster7	Cluster 10	M9(OFF)
G5	1	7.4	98.8	31±5	0.28	0.95	489	0.23	B2	Mon.cluster4	Cluster 1	M1
G6	3	12.6±2.9	195.4±8.5	74±2	0.11±0.01	0.29±0.11	1665±246	0.51±0.03	B3_i_	Mon.cluster9	Cluster 4	M8
G7	4	9.9±0.4	164.4±2.0	19±5	0.15±0.01	0.30±0.13	1347±166	0.42±0.03	B3_o_	Mon.cluster7	Cluster 6	M8
G11	3	13.9±1.6	191.9±6.6	36±2	0.11±0.01	0.26±0.07	1282±131	0.40±0.01	C2_o_	Mon.cluster7	Cluster 10	M9(OFF)
G12	1	14.0	207.7	35±2/66±4	0.16	0.47	2013	0.37				M12
G15	1	6.8	154.5	31±2	0.10	0.91	484	0.25	C6	Mon.cluster6		M5(a)
G17	2	15.1±2.3	143.9±11.3	32±7/63±1	0.17±0.05	0.13±0.09	1011±309	0.38±0.05	D2	Bi.cluster2		M13

Stratification level in the IPL: percent depth from the inner nuclear layer to the ganglion cell layer.

Dendritic arbor density: total dendritic length/dendritic field area.

Asymmetry index: (dendritic length difference between 1st and 3rd quarters + dendritic length difference between of 2nd and 4th quarters)/total dendritic length.

Linear puncta density: total puncta number/total dendritic length.

Values are mean±SEM.

This classification scheme of mouse ganglion cells is based on Völgyi et al., 2009, and is equivalent to (I) Sun et al., 2002; (II) Badea and Nathans, 2004; (III) Kong et al., 2005; (IV) Coombs et al., 2006.

### The synaptic spacing of excitatory inputs varies little across diverse RGCs

**Figure 3:**
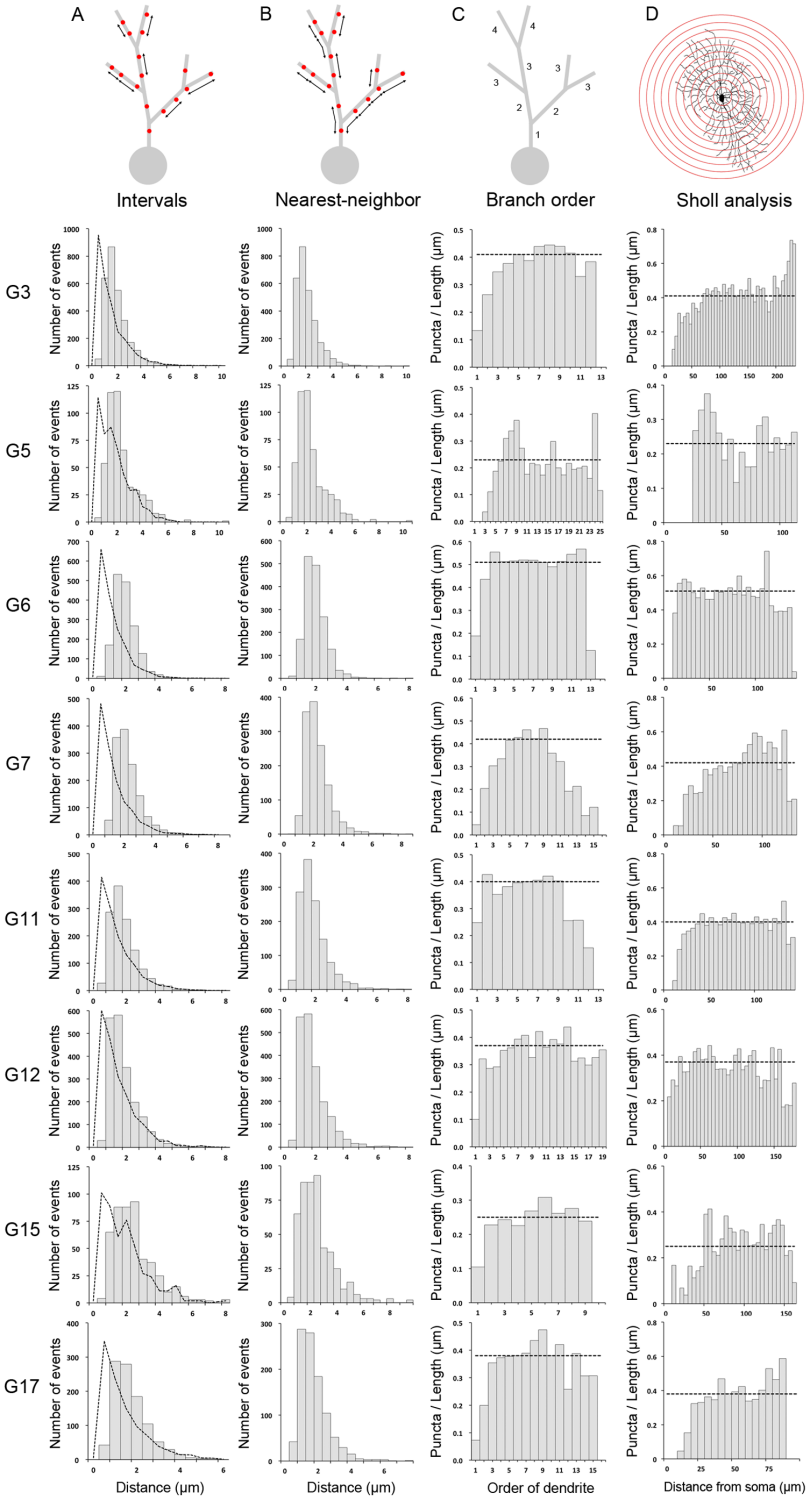
Spatial patterns of excitatory inputs are similar across different types of ganglion cells. (A) The distribution of intervals between puncta. In the total distribution of intervals, it appears that all eight types of ganglion cells have their highest frequency at about 1–2 µm. The actual distribution of PSD95 puncta is compared with the distribution of the same number of puncta on that same dendritic structure at random (dotted lines). (B) The distribution of nearest-neighbor puncta distance. It is apparent that the excitatory synapses are excluded from a reference synapse site at the distance around 1–2 µm for all ganglion cell types. (C) The density of excitatory synaptic inputs as a function of dendritic branch order. In general, there are few synaptic inputs on the first several orders (or, the proximal zone of the dendritic field), and then the linear density of puncta gradually increases and reaches a plateau in the distal zone of the dendritic field in some but not all ganglion cell types. (D) The density of excitatory synaptic inputs as a function of distance from the soma (or, the Sholl analysis). For all cell types, the linear density of puncta near the soma is low. However, the puncta density, once outside the proximal zone, is relatively homogenous across the dendritic field in some but not all ganglion cell types. Note if more than one cell of a cell type has been sampled, the average value is plotted, but the error bar has been omitted for clarity.

Similar to the observation in the rabbit retina [23], the PSD95 puncta were also found to be regularly spaced along the dendritic arbors of mouse ganglion cells, regardless their functional diversity. This impression was verified by two analyses across the different cell types, the interval between puncta and the nearest-neighbor distance. The distribution of the interval between puncta was found to peak at 1–2 µm for all eight ganglion cell types (Fig. 3A). This invariant synaptic spacing would seem to be largely independent of the dendritic field size and the stratification of the cell in the IPL. Examining the distribution of the nearest-neighbor distances of PSD95 puncta also revealed a similar trend, namely that each punctum is surrounded by a 1–2 µm clearance zone that excludes the presence of other puncta across all eight ganglion cell types (Fig. 3B). These results support the hypothesis that the local spatial patterns of glutamatergic inputs in diverse types of mouse ganglion cells are similar, and that the excitatory synapses are regularly spaced at 1–2 µm intervals along the dendrites.

### Spatial distribution of excitatory synapses along the dendrites in various RGCs are similar

To take the dendritic morphologies of different ganglion cell types into account, the spatial distribution of excitatory synapses and their corresponded dendritic structures were examined. Consistent with the analysis of rabbit ganglion cells [2], [23], the distribution of PSD95 puncta also showed a similar trend across the different types of mouse ganglion cells. When the linear puncta density was plotted as a function of dendritic branch order, it is apparent that once outside the proximal zone, the density of synaptic inputs gradually increases and reaches a plateau for most cell types (Fig. 3C), which suggests that the spatial distribution of excitatory synapses does not depend on the branch order of the ganglion cell, although some cells appear to show a decrease in puncta density on the higher order branches (e.g., G5, G7, and G11 cells). Similarly, when the linear puncta density was plotted as a function of distance from the soma (the Sholl analysis), it is evident that the density of excitatory synaptic inputs near the soma is low, but it is relatively homogenous across the dendritic field once the puncta are outside the proximal zone of most ganglion cell types (Fig. 3D). These analyses support the idea that the spatial densities of excitatory synaptic inputs along the dendrites are similar in diverse types of mouse ganglion cells.

### Large cells receive excitatory synaptic inputs more efficiently than small cells

**Figure 4:**
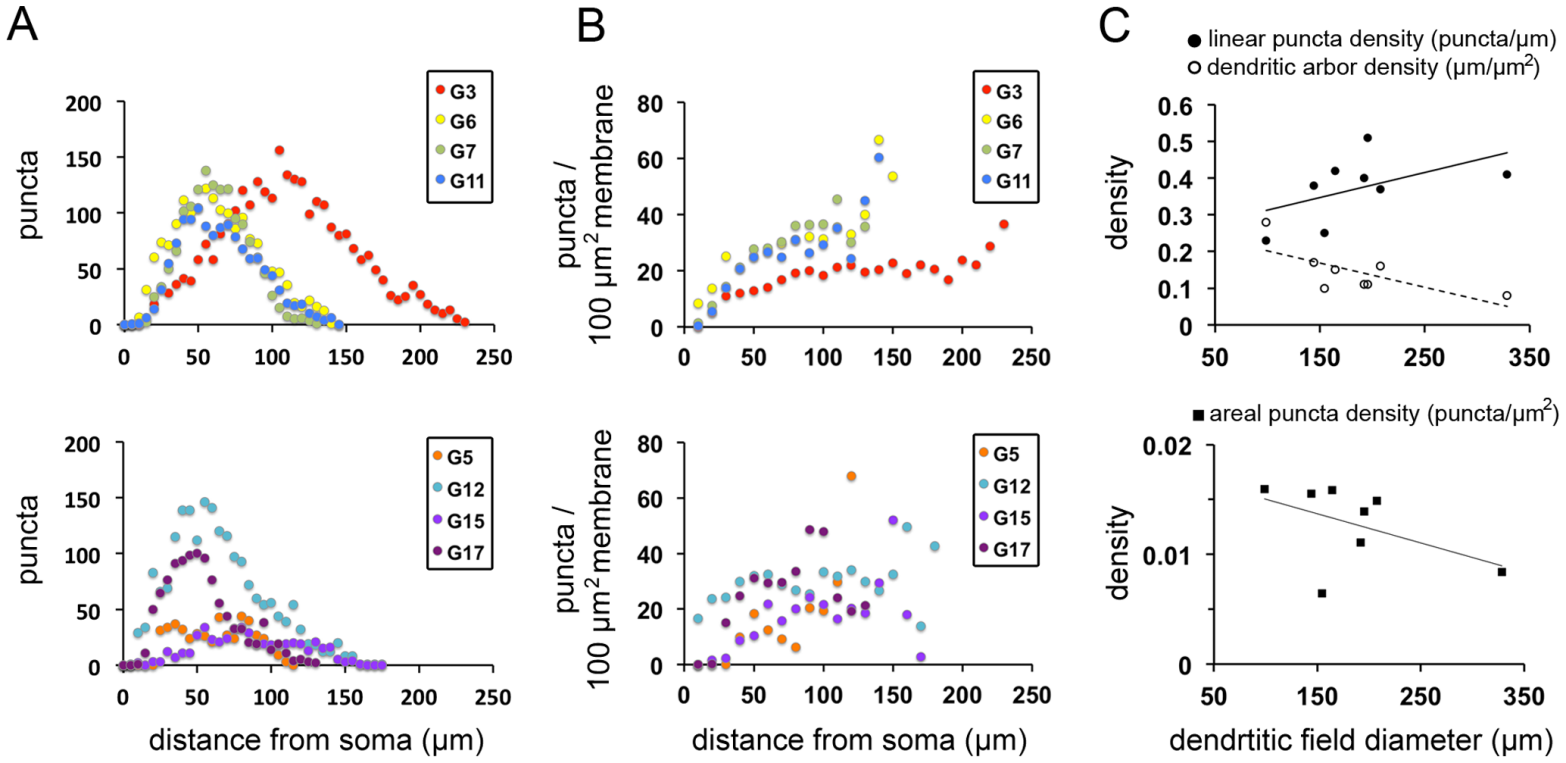
Cells with a larger dendritic field receive excitatory synaptic inputs more efficiently than those with a smaller dendritic field. (A) The plot of the PSD95 puncta numbers as a function of distance from the soma for eight different ganglion cell types. For clarity, four ganglion cell types with similar dendritic patterns (G3, G6, G7, and G11) were plotted in the upper panel, and the rest of cell types were in the lower panel. All cell types show a peak of puncta numbers at a location slightly displaced from the soma. (B) The plot of puncta number per membrane area as a function of distance from the soma for the same ganglion cells. There is a trend of increased puncta/membrane at distal dendrites. (C) The scatter plot of linear puncta density, dendritic arbor density (upper panel), and areal puncta density (lower panel) of each ganglion cell and its dendritic field diameter. Although the dendritic arbor density decreases when the dendritic field size increases, the linear puncta density is slightly higher for cells with a larger dendritic field size. Note if more than one cell of a cell type has been sampled, the average value is plotted, but the error bar has been omitted for clarity.

To compare the spatial distribution of excitatory synapses in ganglion cells with different dendritic field sizes and structures, the PSD95 puncta number was plotted as a function of distance from the soma across the eight different ganglion cell types. Despite a large difference among these cells, the number of excitatory synapses was found to peak at some distance away from the soma and to drop on the distal dendrites (Fig. 4A). This suggests that the excitatory synapses are concentrated at the middle of the dendritic field. The PSD95 puncta number per dendritic membrane area as a function of distance from the soma for the same ganglion cells was also plotted (Fig. 4B). It is apparent that most cells had a tendency of increased puncta/membrane at distal dendrites. This suggests that excitatory synapses are actually more efficiently distributed at periphery than at middle of the dendritic field. Although it is not surprising that large cells have much higher puncta numbers than small cells, it is important to note that the linear puncta density is higher in ganglion cells with a large dendritic field than those with a small dendritic field (Fig. 4C). Similar to the analysis in Jakobs et al. [2], we also found that the area1 puncta density decreases as the dendritic field size increases. However, the linear puncta density is higher for cells with a larger dendritic field size, even though the dendritic arbor density decreases when the dendritic field size increases. This indicates that RGCs with a large dendritic field show increased connectivity of their excitatory synapses to make up for their reduced dendrite density.

## Discussion

Diverse types of mouse ganglion cells were examined in the present study in order to determine whether there were similar spatial relationships of adjacent PSD95 puncta on the dendritic trees of the different types of cell. Such an observation would suggest that there is a common rule for specifying the location of excitatory synaptic inputs along each dendritic arbor, regardless their dendritic structures. The above was found to be true and, in addition, it was also shown that the spatial distribution of excitatory synapses correlates well with the dendritic field size, such that the excitatory synaptic inputs from the distal dendrites of large cells are proportionally increased in order to make up for their reduced dendrite density.

### Isotropy of synapses on the dendrites of RGCs

Previous studies on hippocampal neurons have shown that the density of excitatory inputs is anisotropic around the dendrites, with a higher synaptic density in the thick distal dendrites and a much lower synaptic density in the proximal ones [45]–[47]. However, it has been shown that the situation in the rabbit retina is different to that of hippocampal neurons. It was found that, in the rabbit retina, the excitatory synapses are evenly distributed along most of dendritic arbors, and that this feature is universal across a diverse range of ganglion cells types [2]. In the present study, we explored the situation in the mouse retina and show that the synaptic spacing and distribution are also isotropic around the dendrites across all types of ganglion cells and are independent of cell size, dendritic morphology, and stratification in the IPL (Fig. 3). This common rule of spatial distribution of excitatory synapses on the dendrites of postsynaptic ganglion cells also reflects the output pattern of presynaptic bipolar cells. Since the axon terminals of each bipolar cell type are known to tile the retina and form a regular mosaic [48], [49], the spatial pattern of PSD95 puncta on the dendritic tree of ganglion cells may be constrained by the synaptic outputs of bipolar cells. Whether the presynaptic components of bipolar cells could exert some influence on where the synapse is formed on the dendrite of ganglion cells remains to be investigated.

In addition to the difference in spatial pattern of the excitatory synapses along the dendritic arbors of hippocampal neurons compared to the rabbit and mouse retinas, it has also been found that the ratio of inhibitory input to excitatory input varies significantly around the dendrites in hippocampal neurons [45]. Since the spatial distribution of inhibitory synapses determines en route shunting of excitatory currents being transmitted toward the soma, it was logical to examine the spatial pattern of inhibitory inputs along the dendrites as well as their spatial relationship with the excitatory synapses. Although the plasmids carrying the gene for postsynaptic density of inhibitory synapses, gephyrin, have been delivered to RGCs by particle-mediated transfer [20], the instability of gephyrin expression in retinal neurons precludes its practical use in the present study. A recent developmental study of synaptogenesis using transgenic mice, in which RGCs expressed Neuroligin 2 (NL2), a marker of inhibitory synapses, has shown that this latter approach produces reliable labeling of inhibitory inputs [50]. Future studies using a similar approach for expressing NL2 and PSD95 together will allow us to characterize the spatial distribution of inhibitory and excitatory synapses around the dendrites in a range of mouse ganglion cells at the same time.

### Increased linear density of excitatory synaptic inputs in large RGCs

The ganglion cells characterized in the present study show large differences in the dendritic field size. However, their two-dimensional patterns of excitatory synapses all showed a doughnut-shaped distribution (Fig. 4A), and when these excitatory inputs are integrated at the soma (i.e., the cumulative PSD95 puncta number), the profile in terms of synaptic strength is similar to the Gaussian shaped receptive field center of typical RGCs (data not shown). To take the dendritic membrane area into account, we also analyzed the synaptic density on the membrane across the dendritic arbor for all ganglion cells, but our results are somewhat different from the observation of Xu et al. [18] in guinea pigs, in that we found a trend of increased puncta/membrane at distal dendrites in most cells, although some variations exist (Fig. 4B). This apparent discrepancy may result from the species difference and/or the reduced membrane area in distal dendrites of mouse ganglion cells. Nevertheless, this finding suggests that excitatory synapses may be more efficiently distributed at periphery than at middle of the dendritic field in the mouse retina.

Considering the large difference in the dendritic field size among the different types of RGCs, it is conceivable that the receptive fields of small cells may receive more excitatory inputs per dendritic area than the receptive field of large ones, such that the signal strength can be maintained at the soma for small cells. This is exactly what Jakobs et al. [2] found in rabbits, where they showed that the total density of PSD95 puncta is inversely correlated with the area of the dendritic field in the retina. We also observed a similar trend in the mouse ganglion cells (Fig. 4C). However, when we plotted the linear puncta density as a function of the dendritic field size, the average density of excitatory inputs along the dendritic arbors is higher in large cells than in small ones (Fig. 4C). Since larger cells tend to have sparser dendritic trees and small cells have denser dendritic trees, this results in an inverse relationship between the dendritic arbor density and dendritic field size (Fig. 4C). To maintain similar strengths of signal transfer from the distal dendrites to the soma in large cells, the excitatory inputs along the dendritic arbors need additional boosts to make up for their reduced dendrite density.

In summary, the present study confirms previous observations obtained by examining other mammalian retinas whereby ganglion cells are suggested to be electrotonically compact [2], [18], [23], and they simply sum the excitatory inputs, perhaps also the inhibitory inputs, from the entire dendritic tree. This also implies that the visual signals in the retina are processed at the presynaptic level via the interaction of bipolar and amacrine cells [51]. Although RGCs appear to be compact neurons that do not achieve dendritic integration via spatial patterning of excitation and inhibition, some active properties of dendrites and their resulting nonlinearity may still play significant roles in shaping the trigger features of the cells [52], [53]. Mapping the spatial distribution pattern of excitatory synapses on RGCs thus provides explicit structural information that is essential for our understanding of how excitatory glutamatergic inputs shape neuronal responses.
